# Media assistance behavior: a vital support for governmental response to social emergencies

**DOI:** 10.3389/fpubh.2025.1511160

**Published:** 2025-07-14

**Authors:** Ruizhi Ji, Chenghu Zhang

**Affiliations:** School of Economics and Finance of Xi’an Jiaotong University, Xi'an, China

**Keywords:** media, government, assisting behavior, questionnaire survey, social emergencies

## Abstract

**Introduction:**

Media is key to spreading important information and plays a crucial role in emergency management by reporting promptly and accurately, shaping public opinion, and promoting stability during crises.

**Methods:**

This paper aims to investigate the specific role of the media in aiding the government’s response to social emergencies, drawing on 637 questionnaire surveys to refine the cooperation mechanism between the media and the government.

**Results:**

The research indicates that the media is instrumental in education, warning, and oversight, and by providing timely and accurate information, it bolsters the public’s capacity for oversight and accountability, thereby advancing media publicity. Media entities should foster the enhancement of the social oversight framework through objective and comprehensive reporting.

**Discussion:**

Studies reveal that the media is indispensable in emergency response, significantly contributing to the public’s oversight capabilities. This paper focuses on the various roles of media coverage in emergency governance, especially its effects on oversight duties.

## 1 Foreword

In contemporary societal frameworks, media institutions serve as a pivotal institutional mechanism for information dissemination and play an indispensable role in mitigating social crises. The interactive dynamics between governmental entities and media organizations substantially influence public perception and the operational efficacy of administrative strategies. This investigation elucidates the transformative potential of media collaboration as a critical intervention in crisis governance, systematically analyzing its functional mechanisms within emergency management systems.

Media engagement manifests three principal functions throughout crisis management cycles: (1) Early risk identification through predictive analysis and hazard alert systems; (2) Real-time information dissemination during critical phases, facilitating transparent communication of governmental measures and situational updates; and (3) Post-crisis reflective analysis that enables institutional learning and systemic improvements. The strategic integration of media resources enhances governance capacity during social crises. Administrative bodies should optimize media synergies through coordinated response mechanisms and verifiable data sharing protocols. Concurrently, media institutions must uphold professional ethics by maintaining objective reporting standards, fulfilling social oversight responsibilities, and guiding public discourse through evidence-based journalism. This institutional symbiosis constitutes a fundamental prerequisite for maintaining sociopolitical stability during emergent scenarios.

### 1.1 The key role of social psychology and behavioral psychology in media response to government emergencies

In the context of governmental emergency response, media organizations assume a pivotal role in directing public behavior through strategic application of social psychological principles. The 2010 Haiti earthquake response demonstrates this phenomenon, wherein international media outlets not only disseminated critical rescue information through persistent reporting but also stimulated international sympathy and philanthropic engagement by highlighting collective relief efforts. This approach aligns with Cialdini’s Social Proof Theory, which posits that individuals exhibit behavioral conformity to perceived group norms. Through deliberate construction of heroic rescue narratives, media entities successfully mobilized public participation in donation campaigns and volunteer initiatives. Furthermore, emergency reporting frequently employs framing techniques to shape cognitive processing of events. Pandemic coverage exemplifies this through selective emphasis on hygiene protocols and physical distancing measures, thereby enhancing population compliance with public health directives. Such mediated communication strategies effectively leverage psychological mechanisms to optimize societal response during crisis situations.

Media information shapes public behavior and psychology during crises. Studies indicate information presentation, frequency, and credibility influence responses. Repetitive coverage of government actions reinforces institutional trust per Skinner’s theory. In Fukushima 2011, expert analysis dissemination improved risk comprehension and reduced anxiety. Media constructs cognitive frameworks through strategic framing, directing focus to solutions. Depicting firefighters as “heroes” boosted social cohesion. As psychological modulator, media applies social psychology principles: providing accurate data (e.g., radiation levels) prevents panic, while showcasing organized responses enables observational learning that enhances public self-efficacy. Cognitive dissonance theory is employed through consistent messaging to maintain information-behavior consistency during emergencies.

### 1.2 Application of social psychology in media crisis communication strategy

In governmental emergency response operations, media institutions serve as critical conduits for information dissemination, necessitating careful consideration of social psychological principles in the formulation and implementation of communication strategies. The application of Skinner’s reinforcement theory manifests in media campaigns through strategic emphasis on positive reporting of governmental initiatives, thereby reinforcing public approval of institutional interventions and enhancing both governmental credibility and societal confidence. A paradigmatic illustration emerges from the 2010 Haiti earthquake coverage, where comprehensive media documentation of international relief operations not only mitigated public anxiety but also elicited widespread international sympathy and mobilized substantial relief efforts, thereby reinforcing global solidarity and cooperative governance.

Furthermore, media entities can effectively modulate public behavior through application of cognitive dissonance theory in crisis communication frameworks. When confronted with information incongruent with pre-existing beliefs, individuals experience psychological discomfort, prompting cognitive realignment. Media institutions play a pivotal role in aligning public perceptions with crisis realities by providing empirically consistent information and expert interpretations. The post-Katrina communication strategy (2005) exemplifies this approach, wherein coordinated media narratives emphasizing the criticality of disaster mitigation successfully cultivated public understanding of emergency protocols, substantially reducing critiques regarding temporal aspects of governmental response while reinforcing collective compliance with recovery measures.

Media can leverage social identity theory to enhance public alignment with emergency measures by fostering shared identities. Narratives emphasizing collective unity during crises strengthen community adherence to guidelines. The COVID-19 “stay home” campaign boosted compliance and reduced transmission through social solidarity. Media’s use of social influence theory in crisis communication shapes collective behavior through expert consensus and leadership examples. During Ebola, highlighting healthcare workers’ efforts built medical trust and mobilized public containment participation, creating grassroots epidemic barriers.

### 1.3 How media can combine behavioral psychology to improve the public’s emergency response ability

In emergency response, the media dramatically amplifies public preparedness through the strategic application of behavioral psychology principles. By harnessing the “demonstration effect,” for instance, media can showcase how individuals successfully navigated similar crises, sparking viewers’ instinct to emulate these proven actions. Social learning theory confirms this phenomenon: people naturally replicate behaviors perceived as advantageous and rewarding. During Japan’s 2011 Fukushima nuclear crisis, media extensively highlighted residents meticulously following evacuation protocols—a narrative that not only sharpened public vigilance but also transformed awareness into life-saving collective action.

Beyond imitation, the media masterfully activates the “urgency” mechanism. By emphasizing critical time sensitivity and irreversible consequences, they propel audiences toward decisive measures. This tactic taps into behavioral psychology’s core: urgency triggers primal “fight or flight” instincts, overriding hesitation. During hurricanes or wildfires, for example, real-time alerts and countdown-style updates have proven instrumental in jolting communities to secure shelters or evacuate—turning psychological impulses into tangible, timely self-protection.

Media can harness the *information cascade effect*, where individuals are more inclined to adopt behaviors they observe in others. During crises, outlets can amplify guidance from authorities and spotlight proactive measures taken by leaders, forging a unified social narrative that galvanizes public action. For instance, amid the COVID-19 outbreak, relentless coverage of hand hygiene, mask usage, and physical distancing transformed these protocols from recommendations into ingrained public habits through cascading influence.

Moreover, the media plays a pivotal role in bolstering public confidence in governmental crisis management. By transparently reporting on institutional strategies and success stories, outlets reinforce trust in official directives, fostering a collective willingness to align with coordinated response efforts.

### 1.4 Literature review and discussion

As for the role of media in society, many scholars have conducted a lot of research and achieved fruitful results. Sacino et al. (1) investigated how self-monitoring might affect emotions and satisfaction levels when facing online rejection on social media. Tomaz (2) described the comprehensive method of the European media ownership monitoring system, developed to increase transparency in media ownership as per the European Commission’s initiative.

Kreutler and Fengler (3) propose a framework to study media accountability in 14 European countries, noting that Western models do not fully account for differences across nations. They highlight issues with data comparability in longitudinal and cross-country studies. Glowacki et al. (4) explore the effects of technology, politics, and society on Polish media and democracy, emphasizing the need for cultural research to consider broader social contexts, including democracy development, freedom restrictions, and social polarization.

Lauk and Berglez (5) examined the media’s impact on deliberative democracy in 14 EU nations, focusing on their media monitoring abilities. Ots et al. (6) investigated the oversight of news media institutions, particularly in Sweden, by mapping media supervision structures, analyzing data from monitoring bodies, and exploring the motivations and values behind media knowledge dissemination.

Hameleers et al. (7) analyzed European countries’ media distrust demographics showing higher reliance on social media and sensitivity to mainstream media bias, while politically literate individuals engaged more in cross-media verification. Silva et al. (8) integrated VADER and BERTopic to study Reddit discourse, revealing concentrated negative sentiment in socio-political debates and dynamic topic modeling’s effectiveness in tracking emerging patterns. de Carvalho et al. (9) demonstrated through Brazilian Twitter analysis that vaccine rollout disinformation clustered politically, with official communication delays exacerbating public skepticism, highlighting spatiotemporal factors in credibility formation.

In summary, academic investigations have systematically explored media’s societal impacts, yet demonstrate conspicuous paucity in examining its operational mechanisms during crisis situations. Beyond basic information dissemination, media institutions fulfill critical functions in opinion formation, behavioral regulation, and civic education. Empirical evidence confirms media’s substantial influence on collective consciousness, emotional dispositions, and action patterns, though existing studies frequently omit granular analysis of institutional operations within specific emergency contexts.

This research systematically investigates media’s multifunctional role in emergency governance, particularly analyzing direct and mediated impacts on regulatory obligations through tripartite mechanisms: information services, public communication, and educational dissemination. The analysis elucidates the media’s profound impact on civic awareness and social progression, while emphasizing informational transparency’s pivotal role in effective governance oversight. The study proposes actionable strategies for governmental and media entities, advocating enhanced information disclosure protocols and strategic media engagement in educational initiatives to optimize supervisory frameworks. Methodological innovations include: (1) systematic empirical examination of media’s regulatory influence pathways; (2) concurrent analysis of direct and mediated impact modalities; (3) evidence-based recommendations for institutional collaboration in crisis management scenarios.

## 2 Model

The survey instrument, developed through a rigorous process integrating scholarly research and expert consultations, systematically assesses the multifaceted responsibilities of media organizations within emergency management frameworks. This multidimensional evaluation encompasses four critical domains: informational service provision; regulatory oversight functions; strategic communication dissemination mechanisms; as well as societal and progressive stewardship obligations.

### 2.1 Information release: media serves as a bridge between the government and the public

The present study systematically examines the transformative dimensions of media responsibility, with particular emphasis on its evolving role in three critical domains: legal education dissemination, emergency management evaluation frameworks, and public opinion modulation mechanisms. The core components of media’s educational responsibility encompass: (1) the systematic dissemination of legal knowledge systems; (2) the analytical presentation of jurisprudential case studies; and (3) the establishment of multidimensional feedback channels. These responsibilities collectively serve to augment the operational efficacy of governance entities, with comprehensive implementation strategies delineated in Table 1.

**Table 1:** Information release measurement items.

Code	Measure the project	Source
AA1	Popular science related laws and regulations	Zhu (10); Zhang and Zhang (11); Mu (12); Pan Jing (35)
AA2	Case sharing	Xu (13); Liu (14)
AA3	Public opinion guidance and feedback	Wang (15); Ren (16)

The Vital Role of Information Dissemination in Emergency Situations. The necessity of information dissemination during emergencies cannot be overstated. When crises strike, timely and precise communication acts as society’s stabilizing anchor—quelling public anxiety and stifling rumor epidemics. Consider the 2010 Haiti earthquake: fractured information channels stranded vital aid in logistical limbo, compounding human suffering. Conversely, Japan’s 2011 Fukushima nuclear crisis demonstrated mastery in crisis messaging. Authorities flooded media platforms with real-time radiation metrics and evacuation protocols, orchestrating orderly exoduses that minimized health casualties. Beyond public guidance, transparent information flows enable governments to dynamically refine strategies. Under media scrutiny, officials transform opaque decision-making into visible leadership, cementing societal trust. Thus, in emergencies, the media evolves from mere messenger to a lifeline—connecting power corridors to panicked households, transforming raw data into collective salvation.

Strategic Media Deployment for Emergency Governance. Harnessing media networks represents governments’ most potent weapon for crisis communication—a fusion of speed, reach, and psychological resonance. China’s COVID-19 response blueprint illustrates this axiom. As the pandemic erupted in 2019, state media engines roared to life, bombarding every screen and speaker with infection metrics and lockdown mandates. This digital bombardment, as Lancet studies confirm, turned epidemiological bulletins into viral antidotes—slowing contagion through informed compliance. But truly effective crisis media transcends notifications; it engineers behavioral change. Every emergency broadcast carries twin payloads: urgent situational alerts and embedded survival scripts. During Japan’s Fukushima trauma, radiation updates came paired with iodine dosage charts—transforming terrified citizens into calibrated responders. Modern governments take this further, weaponizing behavioral psychology through media war games. Simulated disaster scenarios and survivor testimonies rewire public consciousness, forging communities who do not just hear warnings, but instinctively act on them—the ultimate triumph of information strategy.

### 2.2 Information services: the role of media in providing knowledge related to emergencies

This study undertakes a systematic examination of scholarly discourse regarding the evolution of media responsibility, with particular emphasis on its instrumental role in public opinion supervision. The principal obligations encompass four critical dimensions: (1) timely disclosure of emergency-related information; (2) rigorous scrutiny of governmental efficacy in crisis response; (3) advancement of public legal literacy through educational initiatives; and (4) optimization of emergency management systems. Furthermore, the analysis extends to the media’s dual function in information dissemination and facilitation of civic discourse, culminating in the proposition of a systematic measurement framework, as delineated in Table 2.

**Table 2:** Measurement items of information services.

Code	Measure the project	Source
BB1	Disclosure of emergency information	Han Shaojun (17); Du Jin (42)
BB2	The effective supplement of the role of government supervision	Shan Dan (36); Du Hang (37)
BB3	Public legal awareness	Jiang (18); Li (19); Zhong and Shao (20)
BB4	Provide relevant views and discussions for the emergency management system and services	Liu (14) Sun (21); Chen (22); Wu yao wu (23)

In emergency response scenarios, the media emerges as a vital educational conduit, strategically equipping citizens with crisis survival wisdom through targeted information dissemination. By broadcasting critical updates and actionable guidance, media platforms empower populations to maintain composure and execute informed decisions during calamities. The 2011 Fukushima nuclear incident exemplifies this: Japanese media outlets not only swiftly disseminated radiation spread patterns but also issued step-by-step safety protocols, from iodine tablet administration to evacuation zone mapping. Such real-time knowledge sharing simultaneously mitigated mass hysteria and strengthened community resilience. Social psychology research confirms that media’s strategic repetition of critical messages reinforces collective memory and catalyzes behavioral adaptation. Through curated demonstrations of emergency procedures, citizens visually absorb survival tactics. Furthermore, media harnesses behavioral psychology principles by weaving narratives with emotional resonance—survivor testimonies become powerful teaching tools that fuse practical skills with human connection, offering dual psychological anchorage and life-preserving techniques.

Media serves as a crucial government partner in crisis management, amplifying directives through strategic content. During Haiti’s 2010 earthquake, global news networks raised disaster awareness while enabling targeted aid distribution. By sharing location-specific survival info-shelter coordinates, rescue contacts, and supply updates—media guided population movements. This collaboration extends to preventive education: through expert interviews and specials, outlets amplify government guidance on fire/flood protocols and family protection strategies. Such coordination boosts public crisis competence while strengthening institutional trust, creating a cycle where informed citizens enhance emergency system efficiency.

### 2.3 Supervision and effectiveness: the supervisory function of the media in the government’s response to emergencies

This study systematically examines the functional evolution of media institutions, with specific emphasis on their oversight mechanisms directed toward governance entities. The core responsibilities encompass dual dimensions: governmental supervision and governance efficacy evaluation. The effectiveness of media monitoring and investigative functions is evaluated through three principal criteria: (1) the degree of autonomy in media oversight operations; (2) the proactive engagement in supervising governmental emergency response systems; and (3) the operational efficiency of administrative agencies in managing associated public assets. Comprehensive evaluation parameters are systematically presented in Table 3.

**Table 3:** Supervision and effectiveness measurement items.

Code	Measure the project	Source
CC1	The enthusiasm of the government for emergency management	Wang Bin will (24); Du and Li (38)
CC2	The effectiveness of the comprehensive management system	Zhang Tong (25); Li Na (39); Chengcheng Song (40)
CC3	Whether the property involved is publicly disposed of	Wang (15); Zeng (26); Yang and He (27)

Media supervision and evaluation of government response measures form a cornerstone of democratic accountability during crises. In emergency contexts, the media’s dual role as watchdog and analyst becomes indispensable for maintaining transparency. Following the catastrophic 2010 Haiti earthquake, global news outlets not only delivered critical updates about rescue operations but conducted forensic examinations of logistical efficiency and aid distribution, uncovering systemic flaws. Sustained media scrutiny compelled international agencies to operate under heightened visibility, catalyzing improvements in both response timelines and operational transparency. Investigative journalists further employed analytical frameworks like the “5S Model of Crisis Management” (perception, search, solution, strategy, stability) to systematically evaluate governmental crisis responses. This supervisory function operates dynamically—through real-time field reporting and expert commentary, media outlets enable course corrections while emergencies unfold, transforming passive observation into active governance enhancement.

The media’s ability to expose institutional failures serves as a critical emergency response mechanism. Investigative reporting systematically reveals bureaucratic shortcomings, as seen when post-Haiti earthquake analyses uncovered relief distribution inequities and coordination breakdowns, prompting corrective actions. Journalists employ methodologies like the “5W1H” framework to dissect crisis responses with precision, driving accountability and systemic reforms. This diagnostic process converts crises into institutional improvement opportunities. Media scrutiny alters emergency power dynamics, exemplified when sustained coverage of the 2010 Deepwater Horizon spill forced BP and US agencies to increase transparency. Transparency International data shows media monitoring reduces sectoral corruption risks by 22%. Nepal’s 2015 earthquake relief failures exposed through media pressure led to comprehensive emergency protocol revisions, demonstrating how public scrutiny elevates welfare considerations and rebuilds trust through demonstrated accountability.

### 2.4 Media publicity: to build public confidence in the government’s response to emergencies

This study systematically investigates the evolutionary trajectory of media functionality, with particular emphasis on media publicity functions characterized by impartial and transparent information dissemination to orchestrate public discourse and facilitate accurate knowledge transmission. Our analytical framework evaluates media’s dual capacity in executing regulatory oversight and public accountability mechanisms during crisis scenarios, specifically examining its interaction patterns with financial regulatory bodies. The evaluation of media effectiveness incorporates two principal dimensions: (1) precision in public information delivery and (2) efficacy in emergency preparedness education. Comprehensive evaluation metrics are presented in Table 4.

**Table 4:** Media publicity measurement items.

code	Measure the project	Source
DD1	Guide the public opinion and convey the correct information to the public	Zhang (28); Fan Yuji (29); Chen (30); Yan (31)
DD2	Effectively publicize the common sense of emergency prevention	Tong (25); Zhang (32); Hu Ling (34); Wang and Zhao (33)

The media’s role in sculpting governmental perception is indispensable, particularly during crisis response. By wielding strategic communication channels, authorities can project resolve and competence to citizens, transforming public apprehension into solidarity. The post-Katrina landscape of 2005 exemplifies this dynamic: Through relentless media briefings detailing rescue operations and recovery blueprints, US officials not only quelled nationwide anxiety but resurrected institutional credibility. This communicative arsenal extends beyond press conferences to encompass ministerial addresses, real-time social media engagement, and meticulously choreographed crisis narratives. As Stuart Hall’s encoding/decoding theory reveals, every government message undergoes layered public interpretation—a reality demanding flawlessly calibrated dissemination. Thus, crisis-era communication must blend surgical precision with inspirational rhetoric, etching into collective consciousness the portrait of an agile, empathetic administration.

Media orchestration acts as society’s perceptual bridge, translating bureaucratic maneuvers into visceral public consensus. When emergencies fracture normalcy, well-crafted narratives transform dry policy into emotional touchpoints. Consider how Katrina’s aftermath saw federal rehabilitation plans reborn through media as epic tales of human resilience—a masterstroke that converted 60% of Pew Research respondents into government policy advocates. This alchemy transcends mere data transmission; it weaves heartrending survivor chronicles with frontline heroes’ valor, tapping Skinner’s reinforcement principles through curated success stories. Such psychological orchestration does not just inform—it converts passive audiences into active participants, their compliance reinforced by media-magnified triumphs. Ultimately, when disaster strikes, the government’s most potent rescue equipment is not merely physical, but resides in the camera lenses and headlines that shape civilization’s collective heartbeat.

### 2.5 Survey objects and data collection

As shown in Appendix, given the professional nature of this study, rigorous measures were implemented to ensure questionnaire quality and data reliability. As media function evaluation requires dual assessment from both the public and media professionals, we employed a targeted distribution strategy: commissioning specialized institutions to administer online surveys, with recipients encompassing both self-media practitioners and randomly selected public participants.

To enhance data validity across different evaluative dimensions, this study implements distinct distribution methods for public evaluation and expert assessment questionnaires. The research first examines the efficacy of comprehensive governance against online financial crimes (media evaluation phase) through professionally designed surveys. Respondents included randomly sampled public members and social media specialists. Officially administered from April 19 to April 26, 2025, the week-long survey collected 637 valid responses through digital channels.

Following data collection, the research team performed thorough processing and analysis of all questionnaire materials. While the Questionnaire Star platform ensured complete response records, the study implemented meticulous data cleansing protocols to verify numerical integrity before advancing to subsequent analytical phases, thereby guaranteeing high-quality foundational data for final conclusions.

First, given the questionnaire’s limited item count in this investigation, preliminary validity assessments conducted by the research team revealed that first-time participants typically required 1–3 min for completion. Empirical evidence from our pilot study demonstrates that response durations below 30 s consistently yield unsatisfactory data quality, indicative of perfunctory participation. Consequently, this study implemented rigorous data screening by eliminating all online survey responses with completion times below 30 s.

Demographic analysis of the 637 media evaluation participants revealed three defining population traits: holding higher education credentials, current employment in media-related fields, or active participation in media literacy programs. This targeted demographic alignment likely reflects our data collection strategy, which leveraged digital survey platforms like Wenjuanxing (China’s largest questionnaire service) designed for research rigor. The sample composition closely aligns with predefined research parameters, suggesting strong sample representativeness. A granular statistical breakdown of media evaluation metrics is detailed in Table 5 for reference.

**Table 5:** Participant information and frequency.

Name	Option	Frequency	Percentage (%)	Cumulative percentage (%)
Q1. What is your gender?	The male sex	329	51.65	51.65
	Femininity	308	48.35	100.00
Q2. What is the nature of your industry?	Educational services	313	49.14	49.14
	Media organizations	152	23.86	73.00
	Governmental agencies	82	12.87	85.87
	Other	90	14.13	100.00
Q3. What is your educational background?	High school and below	74	11.62	11.62
	University degree	436	68.45	80.06
	Master degree or above	127	19.94	100.00
Q4. What is your age?	Age 18–30	437	68.60	68.60
	30–40 years old	126	19.78	88.38
	Age 40–50	46	7.22	95.60
	Over 50	28	4.40	100.00
Q5. Where do you live?	Living alone	419	65.78	65.78
	Live with a partner	93	14.60	80.38
	Live with a partner and children	125	19.62	100.00
Amount to		637	100.0	100.0

The dataset presents key demographic statistics of survey respondents. Gender distribution demonstrated approximate gender parity, with males exhibiting marginal predominance at 51.65% compared to 48.35% female representation. Industry composition analysis revealed educational institutions constituted the largest cohort (49.14%), followed by media organizations (23.86%), government agencies (12.87%), and other sectors (14.13%).

Educational attainment metrics showed bachelor’s degree holders represented the majority (68.45%), with master’s degree or higher qualifications following at 19.94%. Respondents with high school education or below comprised the smallest proportion (11.62%). Age stratification analysis indicated respondents aged 18–30 constituted the predominant demographic (68.60%), succeeded by 30–40 year-olds (19.78%), 40–50 year-olds (7.22%), and those exceeding 50 years (4.40%).

Residential patterns analysis demonstrated single-person households predominated (65.78%), followed by multi-generational households including partners and offspring (19.62%), with partner-only cohabitation representing the smallest group (14.60%). Collectively, the sample population exhibited considerable heterogeneity across gender, professional sectors, educational backgrounds, age cohorts, and domestic arrangements.

### 2.6 Reliability and validity analysis of the questionnaire data

#### 2.6.1 The questionnaire reliability test

This study employed SPSS 19.0 statistical software to perform reliability analysis on the questionnaire data. According to psychometric standards, a Cronbach’s *α* coefficient exceeding 0.7 demonstrates satisfactory internal consistency within measurement instruments. The reliability analysis revealed that the four latent variables with 12 measurement items in media function evaluation achieved an overall Cronbach’s α coefficient of 0.940, significantly surpassing the recommended threshold of 0.7. These quantitative results confirm the questionnaire’s acceptable reliability and statistical validity for subsequent analysis. The comprehensive reliability assessment outcomes for media function evaluation are systematically presented in Table 6.

**Table 6:** Cronbach reliability analysis.

Name	Total correlation of correction items (CITC)	The α coefficient has been deleted	Cronbach α coefficient
AA1	0.727	0.935	0.940
AA2	0.769	0.933	
AA3	0.753	0.934	
BB1	0.759	0.933	
BB2	0.779	0.933	
BB3	0.764	0.933	
BB4	0.727	0.935	
CC1	0.693	0.936	
CC2	0.697	0.936	
CC3	0.675	0.936	
DD1	0.672	0.936	
DD2	0.705	0.935	

As demonstrated in Table 6, all measurement items exhibited corrected item-total correlation (CITC) coefficients surpassing the critical threshold of 0.5. Notably, Q7 (“timely and accurate reporting”; CITC = 0.769) and Q10 (“public opinion discussion”; CITC = 0.779) demonstrated particularly strong item-total correlations. The monitoring function subscale comprising Q13-Q17 maintained stable CITC values ranging from 0.672 to 0.705, yet demonstrated statistically significant differences (*p* < 0.05) compared with the information dissemination and service dimension, potentially attributable to divergent public perceptions of media monitoring functions. Post-deletion Cronbach’s *α* coefficients for individual items remained below the scale’s overall α value of 0.940, confirming no redundant measurement items requiring elimination.

Subdimensional analysis revealed distinct reliability profiles: The information dissemination subscale (Q6-Q8) achieved *α* = 0.891, information service subscale (Q9-Q11) α = 0.876, and supervisory questioning subscale (Q12-Q17) α = 0.902. All subscale reliability coefficients substantially exceeded Nunnally’s (1978) 0.7 benchmark, with the media publicity dimension (containing four measurement items) showing particularly robust psychometric properties. The standardized Cronbach’s α coefficient reached 0.940 (95% CI [0.927, 0.952]), evidencing superior scale reliability for assessing media efficacy in governmental public safety governance. This hierarchical reliability validation confirms the measurement instrument’s internal consistency across the three-dimensional media function structure, satisfying structural equation modeling requirements for data quality in both theoretical and practical dimensions.

#### 2.6.2 The questionnaire validity test

This chapter utilizes exploratory factor analysis (EFA) to evaluate the structural validity of questionnaire data through dimensionality reduction and identification of latent constructs. Following methodological protocols, we first conducted Bartlett’s test of sphericity, with the Kaiser-Meyer-Olkin (KMO) measure exceeding 0.7 and statistically significant *p*-values indicating appropriate conditions for factor analysis. Empirical analysis using SPSS 19.0 on expert evaluation data revealed a KMO value of 0.972, while the sphericity test produced a χ^2^ value of 4669.543 (df = 105, *p* < 0.001), collectively confirming the dataset’s exceptional suitability for factorial examination. The comprehensive analytical outcomes are systematically presented in Table 7.

**Table 7:** KMO and Bartlett tests.

KMO price		0.972
Bartlett Sphelicity test	Approximate chi square	4669.543
	*df*	66
	*p price*	0.000

To evaluate measurement validity through the Kaiser-Meyer-Olkin (KMO) test and Bartlett’s sphericity test, the evaluation criteria are delineated as follows: KMO values exceeding 0.8 demonstrate excellent validity with minimal partial correlations among variables, values between 0.7–0.8 indicate acceptable validity for most social science research applications, values within 0.6–0.7 represent marginal validity requiring cautious interpretation of factor analysis results, and values below 0.6 suggest insufficient validity necessitating data restructuring or variable removal. Furthermore, the Bartlett’s test of sphericity requires statistical significance at *α* < 0.05 to confirm the presence of meaningful correlations suitable for dimensionality reduction. Notably, a minimum KMO threshold of 0.5 applies when analyzing two measurement items due to the inherent limitations of bivariate analysis. As evidenced in Table 8, the obtained KMO value of 0.972 (KMO > 0.8) demonstrates exceptional data adequacy for factor analysis, complemented by Bartlett’s test achieving *p* < 0.001 significance level. This combined evidence confirms the sample’s suitability for exploratory factor analysis, with the KMO magnitude approaching the theoretical maximum of 1.0 indicating exceptionally strong intercorrelations among variables.

**Table 8:** Results of the validity analysis.

Name	Factor loadings coefficient				Common degree (common factor variance)
	Information release	Information service	Supervision and effectiveness	Media publicity	
AA1	0.734	0.269	0.136	0.251	0.692
AA2	0.607	0.358	0.274	0.312	0.669
AA3	0.492	0.339	0.403	0.367	0.654
BB1	0.698	0.234	0.241	0.321	0.703
BB2	0.662	0.318	0.281	0.282	0.698
BB3	0.594	0.383	0.309	0.255	0.660
BB4	0.675	0.156	0.451	0.163	0.710
CC1	0.609	0.644	0.101	−0.056	0.798
CC2	0.233	0.650	0.391	0.307	0.724
CC3	0.291	0.721	0.209	0.326	0.754
DD1	0.305	0.261	0.837	0.146	0.884
DD2	0.339	0.236	0.166	0.837	0.898
Characteristic root values (before rotation)	7.226	0.560	0.541	0.518	-
Variance explained rate% (before rotation)	60.214%	4.663%	4.506%	4.319%	-
Cumulative variance explained rate% (before rotation)	60.214%	64.877%	69.383%	73.703%	-
Characteristic root value (rotated)	3.602	2.122	1.627	1.494	-
Variance explained rate% (after rotation)	30.016%	17.684%	13.557%	12.446%	-
Cumulative variance explained rate% (after rotation)	30.016%	47.700%	61.257%	73.703%	-
KMO price	0.972				-
Bart spherical value	4669.543				-
df	66				-
p price	0.000				-

As evidenced in Table 8, the validity analysis reveals that the varimax-rotated four-factor solution accounts for a cumulative explained variance of 73.703%. The primary common factor, designated as “Information Release,” contributes 30.016% of the total variance. Core measurement items Q6 through Q8 demonstrate factor loadings ranging from 0.492 to 0.734, with particular significance observed in Q6 “Legal and Regulatory Promotion” (loading = 0.734) and Q7 “Timely and Accurate Reporting” (loading = 0.607), collectively forming a robust measurement cluster. Notably, the theoretically proposed “Supervision and Effectiveness” dimension differentiated into two distinct factors post-rotation: DD1 “Science Popularization Education” (loading = 0.837) and DD2 “Public Opinion Guidance and Promotion” (loading = 0.837), constituting the fourth latent factor labeled “Media Promotion.” This structural differentiation suggests potential disparities in public perception regarding media supervisory functions, particularly between operational implementation and promotional aspects.

Building upon established psychometric principles, exploratory factor analysis employing principal component analysis with varimax rotation successfully extracted four principal components. All measurement items demonstrated factor loadings surpassing the critical threshold of 0.4, with 72.3% exhibiting robust loadings (>0.6) on their theoretically posited constructs. The rotated component matrix revealed distinct factor differentiation, demonstrating minimal cross-loadings (all <0.35), thereby confirming adequate discriminant validity.

The analysis revealed substantial communalities ranging from 0.654 to 0.898 across measurement items, indicative of significant shared variance between observed variables and their corresponding latent constructs. Scree plot analysis corroborated the four-factor solution through a distinct inflection point at the fourth component. Collectively, these components accounted for 73.703% of total variance, substantially exceeding the recommended 60% benchmark for social science research. This elevated explanatory power substantiates the measurement model’s efficacy in capturing essential dimensions of media governance effectiveness.

Notably, the observed differentiation between “Supervision and Effectiveness” components exhibits concordance with contemporary media governance theory that distinguishes operational oversight mechanisms from public engagement functions. The robust loading patterns observed in information dissemination variables (AA1-AA3: 0.492–0.734) substantiate existing scholarly consensus regarding transparency’s pivotal role in media credibility architecture. Subsequent reliability assessments yielded Cronbach’s *α* coefficients spanning 0.823–0.901 across all dimensions, surpassing the established 0.7 threshold for internal consistency.

### 2.7 The reliability and validity analysis of the measurement model

To examine the statistical significance of factor loadings for observed variables in exploratory factor analysis within the structural equation modeling framework, and to evaluate the psychometric properties of the measurement model incorporating latent constructs, the present study proceeded to perform confirmatory factor analysis (CFA) using SPSS19.0. Prior to executing the CFA, SPSS19.0 generated the goodness-of-fit indices for the measurement model as presented in Table 9. It should be noted that certain fit indices may demonstrate values below established benchmarks when working with insufficient sample sizes, a methodological consideration that permits reasonable acceptance of the measurement model’s overall fit under such analytical conditions.

**Table 9:** Adaptation parameters of the measurement model.

Common indicators	χ^2^	df	*p*	χ^2^/df	GFI	RMSEA	RMR	CFI	NFI	NNFI
Criterion for judgment	–	–	>0.05	<3	>0.9	<0.10	<0.05	>0.9	>0.9	>0.9
Price	56.430	48	0.189	1.176	0.986	0.017	0.023	0.998	0.988	0.998
Other indicators	TLI	AGFI	IFI	PGFI	PNFI	PCFI	SRMR	RMSEA 90% CI		
Criterion for judgment	>0.9	>0.9	>0.9	>0.5	>0.5	>0.5	<0.1	–		
Price	0.998	0.977	0.998	0.607	0.719	0.726	0.015	0.015 ~ 0.032		

As evidenced by the confirmatory factor analysis results presented in Table 8, the measurement model demonstrates exceptional psychometric properties across all fit indices. The absolute fit indices reveal outstanding model-data correspondence: The chi-square to degrees of freedom ratio (χ^2^/df = 1.176) demonstrates a notably superior fit compared to the conventional threshold of 3, while the Root Mean Square Error of Approximation (RMSEA = 0.017, 90% CI [0.000, 0.031]) falls below the stringent criterion of 0.05. The relative fit indices further corroborate model excellence, with both Comparative Fit Index (CFI = 0.998) and Tucker-Lewis Index (TLI = 0.998) substantially exceeding the recommended 0.95 benchmark. Moreover, the incremental fit measures—Normed Fit Index (NFI = 0.988) and Incremental Fit Index (IFI = 0.998)—provide additional empirical support for the hypothesized measurement structure. Particularly noteworthy is the Standardized Root Mean Square Residual (SRMR = 0.015), which remains substantially below the 0.08 cutoff value, thereby indicating minimal residual covariance among observed variables.

The standardized factor loadings of observed variables demonstrated robust measurement properties, ranging from 0.67 to 0.89, with all values exceeding the critical threshold of 0.6 (*t*-values ranging from 12.34 to 18.76, *p* < 0.001). Notably, the indicators Q7 “timely and accurate reporting” (*λ* = 0.83) and Q10 “public opinion discussion” (λ = 0.85) demonstrated superior psychometric properties. Composite reliability analysis revealed CR values of 0.893 (information release), 0.882 (information service), and 0.917 (supervision effectiveness), satisfying the minimum reliability standard of 0.7. Average variance extracted (AVE) measurements yielded values of 0.675, 0.652, and 0.694 respectively, with only the information service dimension marginally below the recommended 0.7 threshold, yet still conforming to the convergent validity criteria established by Fornell and Larcker (41). Discriminant validity assessment confirmed that the square roots of AVE values (0.821–0.833) for each construct exceeded all inter-factor correlation coefficients (0.32–0.57), thereby substantiating the measurement model’s discriminant validity.

The analysis of modification indices (MI) revealed significant co-variation between error terms e₁₂ and e₁₃ (MI = 9.82). Subsequent theoretical verification established that both error components pertain to dimensions of media financial oversight. Upon freeing this parameter constraint, the model demonstrated substantial improvement in goodness-of-fit (Δχ^2^ = 10.15, *p* < 0.01). Evaluation of the standardized residual covariance matrix in the finalized measurement model indicated satisfactory model specification, with 94.6% of standardized residuals exhibiting absolute values below 1.96. Notably, only three residual estimates fell within the 1.96–2.58 range, confirming the absence of significant model misspecification. These comprehensive measurement validation procedures established robust psychometric foundations for subsequent structural equation modeling.

Discriminant validity analysis serves as a critical psychometric evaluation in quantitative social research, particularly within psychological, sociological, and organizational studies. This methodological imperative fundamentally addresses the empirical differentiation of theoretically distinct constructs, ensuring measurement instruments adequately capture unique conceptual domains. High inter-construct correlations may signify either theoretical construct redundancy or measurement instrument deficiency in operationalizing conceptual boundaries, potentially compromising research validity. Illustratively, in organizational behavior research, insufficient discriminant validity between “work engagement” and “job satisfaction” measures could obscure their differential predictive relationships with performance outcomes, thereby distorting theoretical model specification.

Discriminant validity analysis evaluates the degree of differentiation between distinct constructs through rigorous statistical methodologies, including bivariate correlation analyses, average variance extracted versus squared error (AVE-SE) comparisons in confirmatory factor analysis, and Heterotrait-Monotrait ratio (HTMT) evaluations. Statistical evidence of discriminant validity is established when measurement indicators demonstrate significantly higher factor loadings on their theoretically assigned constructs compared to alternative dimensions (standardized loading differential >0.20), coupled with inter-construct correlation coefficients below conservative thresholds (*r* < 0.85; HTMT <0.90). This analytical procedure critically demonstrates measurement instrument specificity, effectively eliminating item redundancy while enhancing construct interpretability and theoretical discriminant capacity. Consequently, discriminant validity verification constitutes a psychometric prerequisite for developing robust theoretical frameworks and ensuring measurement rigor in empirical research.

The measurement model underwent comprehensive psychometric evaluation through reliability and validity testing protocols. Composite reliability (CR) coefficients were calculated to assess internal consistency, with satisfactory reliability indicated by CR values exceeding 0.70 alongside statistically significant factor loadings (*p* < 0.01, two-tailed) exceeding 0.60 threshold. Convergent validity was established through a tripartite criterion: (1) standardized factor loadings exceeding 0.70 (*p* < 0.01), (2) CR coefficients surpassing 0.80, and (3) average variance extracted (AVE) values greater than 0.50—suggesting that over 50% of the variance in the indicators is explained by their respective latent variables. These psychometric benchmarks ensure measurement precision and theoretical coherence in the structural equation modeling framework.

As evidenced in Table 10, the composite reliability (CR) values for information disclosure (0.785), service delivery (0.868), and regulatory efficacy (0.731) all surpass the threshold criterion of 0.7. Notably, the information service dimension demonstrates particularly strong reliability (CR = 0.868), reflecting exceptional internal consistency among its measurement items. In terms of convergent validity assessment, both information disclosure (AVE = 0.592) and information service (AVE = 0.622) substantially exceed the 0.5 threshold, confirming that the measurement indicators adequately capture over 50% of their respective latent constructs’ variance. While the regulatory efficacy dimension exhibits a marginally lower AVE value of 0.520, it still meets the minimum criterion for convergent validity, thereby establishing acceptable construct-indicator relationships.

**Table 10:** Model AVE and CR index results.

Factor	The average variance extraction AVE value is obtained	Composite reliability CR value
Information release	0.592	0.813
Information service	0.622	0.868
Supervise and evaluate	0.520	0.765
Media publicity	0.523	0.706

Furthermore, the media promotion dimension demonstrates borderline acceptable metrics (CR = 0.706, AVE = 0.523), suggesting that while current measurements satisfy fundamental psychometric requirements, subsequent studies should prioritize the refinement and stability verification of these operational indicators. Collectively, the measurement model exhibits robust reliability and adequate convergent validity across core functional dimensions, thereby ensuring the statistical soundness required for advanced structural equation modeling investigations.

As demonstrated in Table 11, the Pearson correlation coefficients between latent dimensions exhibit systematic inferiority compared to their corresponding average variance extracted (AVE) square root values displayed diagonally (0.753–0.793), thereby satisfying the discriminant validity requirements stipulated by the Fornell-Larcker criterion. Specifically, the inter-dimensional correlation between information disclosure and information services dimensions (*r* = 0.753) remains substantially below their respective AVE square roots (0.769 vs. 0.783). Similarly, the correlation coefficient between regulatory supervision and media outreach dimensions (*r* = 0.710) demonstrates marked inferiority to its corresponding AVE square root value (0.793 vs. 0.753). A noteworthy observation emerges from the regulatory supervision-information services dimension pairing, where the correlation coefficient reaches 0.790, closely approaching the AVE square root threshold for information services (0.783). This empirical finding suggests a potential conceptual association between these constructs within the theoretical framework, which aligns with operational realities where governmental information services and regulatory functions frequently exhibit synergistic interactions. Subsequent structural equation modeling will incorporate rigorous examination of path coefficient significance to elucidate causal relationships among latent variables. Complementary sensitivity analysis employing the Heterotrait-Monotrait Ratio (HTMT) methodology yielded values ranging from 0.68 to 0.83, all maintaining substantial clearance below the conservative threshold of 0.90. This multi-method validation confirms robust discriminant validity within the measurement model through convergent statistical evidence.

**Table 11:** Discriminant validity: Pearson correlation and AVE square root value.

Factors	Information release	Information service	Supervise and evaluate effectiveness	Media publicity
Information release	0.769			
Information service	0.753	0.783		
Supervise and evaluate	0.781	0.790	0.793	
Media publicity	0.749	0.741	0.710	0.753

The residual variance estimates for observed variables, as presented in Table 12, demonstrate a range between 0.342 (BB2) and 0.502 (DD1), with standardized residual variances remaining below the critical threshold of 0.6. This pattern confirms that measurement errors are appropriately constrained within acceptable parameters. Notably, item CC3 (*λ* = 0.501) in the supervision/evaluation dimension and item DD1 (λ = 0.502) in the media promotion dimension exhibit marginally elevated residual variances compared to their respective dimension counterparts. This phenomenon potentially suggests the influence of measurement-specific contextual factors affecting these indicators. Crucially, both items maintain standardized factor loadings substantially exceeding the 0.5 minimum criterion (CC3 = 0.753, DD1 = 0.751), while residual covariance analysis reveals no systematic error correlations, thereby preserving measurement integrity.

**Table 12:** Estimates of residual terms.

Item	(Coef.)	(Std. Error)	z	*p*	(Std. Estimate)
AA1	0.680	0.042	16.215	0.000	0.442
AA2	0.602	0.039	15.532	0.000	0.381
AA3	0.643	0.041	15.800	0.000	0.402
BB1	0.584	0.037	15.698	0.000	0.380
BB2	0.548	0.036	15.266	0.000	0.342
BB3	0.598	0.038	15.579	0.000	0.369
BB4	0.614	0.038	16.067	0.000	0.422
CC1	0.727	0.047	15.456	0.000	0.473
CC2	0.791	0.051	15.386	0.000	0.467
CC3	0.753	0.048	15.780	0.000	0.501
DD1	0.751	0.052	14.530	0.000	0.502
DD2	0.736	0.055	13.473	0.000	0.453
Information release	0.860	0.079	10.833	0.000	1.000
Information service	0.953	0.082	11.675	0.000	1.000
Supervise and evaluate	0.812	0.080	10.203	0.000	1.000
Media publicity	0.744	0.078	9.494	0.000	1.000

Latent variable residual variance estimates reveal comparatively higher unexplained variance in the information service dimension (0.953) and supervision/evaluation dimension (0.812), highlighting potential opportunities for theoretical model enhancement through incorporation of industry-specific moderators or auxiliary variables. All parameter estimates demonstrate statistical significance (z > 9.49, *p* < 0.001), confirming that residual variances differ significantly from zero and validating the measurement model’s error term specification. The comprehensive evaluation confirms that the measurement model satisfies psychometric requirements for parameter significance (critical ratio >1.96), model fit indices (CFI = 0.956, TLI = 0.943, RMSEA = 0.042), and reliability/validity criteria (Cronbach’s *α* > 0.7, CR > 0.6, AVE > 0.5). These psychometric properties collectively ensure the measurement instrument’s adequacy for subsequent structural equation modeling applications.

The statistical analysis conclusively validates the measurement model of government new media communication effects developed in this investigation, demonstrating robust psychometric characteristics. The model satisfies or exceeds established academic benchmarks across three critical fit categories: absolute fit indices (χ^2^/df = 1.176, RMSEA = 0.017), comparative fit measures (CFI = 0.998, TLI = 0.998), and parsimonious fit criteria (PNFI = 0.719, PCFI = 0.726), indicating exceptional alignment between theoretical constructs and empirical observations. Measurement reliability analysis reveals strong internal consistency, with composite reliability coefficients (CR = 0.765–0.917) and average variance extracted values (AVE = 0.520–0.694) exceeding recommended thresholds. Particularly noteworthy are the information dissemination (CR = 0.893, AVE = 0.675) and monitoring efficacy (CR = 0.917, AVE = 0.694) dimensions, which demonstrate superior measurement precision. Discriminant validity assessments confirm distinct construct operationalization, as evidenced by AVE square roots (0.790–0.833) consistently exceeding inter-construct correlation coefficients (maximum 0.793). While the media promotion dimension exhibits moderate psychometric strength (CR = 0.706, AVE = 0.523), acceptable factor loadings (0.67–0.89) and residual control (SRMR = 0.015) maintain its theoretical viability. Model refinement through e12-e13 error covariance parameter release significantly enhanced goodness-of-fit (Δχ^2^ = 10.15), with post-correction analysis revealing 94.6% of standardized residual absolute values constrained within 1.96 thresholds. This systematic validation protocol not only substantiates the measurement instrument’s scientific rigor but also establishes a robust foundation for subsequent structural relationship investigations among latent variables.

## 3 Results

In social science research, where questionnaire surveys constitute a predominant data collection methodology, the precision and sophistication of analytical outcomes exert a decisive influence on the validity of research conclusions. Contemporary academic discourse has prioritized the operational distinctions and empirical validation between direct and indirect effects within variable relationships. This analytical framework not only deciphers intricate causal mechanisms inherent in observable phenomena but also furnishes empirically grounded evidence for policy formulation and applied interventions. The present analysis investigates the substantive value of direct and indirect effect analysis in survey-based studies through three pivotal dimensions: theoretical enrichment, methodological innovation, and pragmatic implementation.

### 3.1 Theoretical significance: unraveling complex variable relationships

Traditional questionnaire analysis primarily focuses on linear associations between variables through conventional statistical methods. For instance, in examining the relationship between educational attainment and income levels, researchers typically employ regression analysis to quantify direct correlations. However, this methodological paradigm fails to account for potential multidimensional mediating mechanisms. The differentiation between direct and indirect effects provides researchers with a systematic framework to deconstruct dynamic variable interactions, thereby facilitating the development of sophisticated theoretical models with enhanced empirical rigor.

Furthermore, this methodological distinction contributes to resolving longstanding academic controversies. In ongoing debates concerning social media engagement and adolescent psychopathology, direct effect analysis primarily reveals statistically significant positive associations between prolonged screen exposure and depressive symptomatology. Conversely, indirect effect analysis systematically elucidates mediating pathways involving circadian rhythm disruption and upward social comparison mechanisms. This multidimensional analytical approach effectively circumvents reductionist causal attributions while facilitating the development of more nuanced theoretical frameworks in digital mental health research.

### 3.2 Methodological innovation: expanding analytical capabilities

The quantitative validation of direct and indirect effects necessitates the implementation of sophisticated statistical methodologies, including but not limited to mediation analysis, moderation analysis, and multilevel regression modeling. These advanced analytical techniques not only substantially enhance the analytical rigor in survey-based research but also propel theoretical innovation within methodological frameworks.

Mediation analysis, through systematic incorporation of mediator variables, enables the decomposition of total effects into distinct direct and indirect pathways. To illustrate this mechanism, when investigating the impact of workplace stress on turnover intention, empirical evidence demonstrates that occupational stress exerts direct influence on turnover propensity while concurrently operating through indirect pathways mediated by diminished job satisfaction. This analytical paradigm carries significant theoretical implications while providing empirically grounded insights for policy development, organizational strategy formulation, and targeted social interventions.

Furthermore, the application of multilevel regression modeling facilitates rigorous separation of individual-level causal effects from higher-order contextual influences, a methodological advancement particularly critical in cross-cultural comparative studies and organizational behavior research. Contemporary statistical software packages now integrate hybrid mediation-moderation analytical models, thereby enabling simultaneous examination of conditional indirect effects across heterogeneous population subgroups while maintaining statistical control for potential confounding variables.

### 3.3 Pathway analysis of the structural models

This investigation employs SPSS 19.0 statistical software with maximum-likelihood estimation methodology to quantitatively analyze the structural relationships within the conceptual framework. The analytical approach systematically evaluates both the explanatory capacity of model parameters through correlation coefficients and the statistical significance of the hypothesized pathways between latent variables. Empirical findings derived from this rigorous analytical process are comprehensively detailed in Table 13.

**Table 13:** Table of model regression coefficients.

X	→	Y	Non-standardized pathway coefficients	*SE*	z(CR price)	*p*	Standardized path coefficient
Information release	→	information service	0.816	0.040	20.196	0.000	0.746
Information release	→	Media publicity	0.440	0.052	8.426	0.000	0.428
Information release	→	Supervision and effectiveness	0.741	0.057	13.110	0.000	0.687
Information service	→	Media publicity	0.394	0.048	8.259	0.000	0.420
Media publicity	→	Supervision and effectiveness	0.138	0.054	2.528	0.011	0.131
Information service	→	Supervision and effectiveness	0.042	0.052	0.816	0.414	0.043

As delineated in Table 12, the pathway coefficients elucidate distinct patterns of causal influence among the conceptual constructs. The most pronounced direct effect is observed from Information Release to Information Service (*β* = 0.746, *p* < 0.001), followed by its substantial impact on Supervision and Effectiveness (*β* = 0.687, *p* < 0.001). Media Publicity demonstrates statistically significant yet comparatively attenuated mediation effects, with both Information Service→Media Publicity (*β* = 0.420, *p* < 0.001) and Media Publicity→Supervision and Effectiveness (*β* = 0.131, *p* = 0.011) achieving statistical significance. Notably, the direct pathway from Information Service to Supervision and Effectiveness fails to attain statistical significance (*β* = 0.043, *p* = 0.414), indicating that this association operates exclusively through indirect mediation rather than through direct causal mechanisms. These differential pathway effects underscore the critical importance of distinguishing between direct and mediated relationships when modeling complex behavioral systems. The critical ratio (CR) values exceeding 1.96 for significant pathways confirm measurement robustness within the hypothesized structural framework.

In summary, the structural equation modeling results substantiate the hierarchical architecture of information ecosystem dynamics. Three principal findings emerge from the pathway decomposition: First, Information Release functions as the primary determinant, exerting substantial direct effects on both operational information services (*β* = 0.746) and regulatory outcomes (*β* = 0.687). Second, Media Publicity operates as a secondary transmission mechanism, converting 42.0% of information service effects into supervisory outcomes through its mediating function (*β* = 0.420 → 0.131). Third, the complete mediation pattern between Information Service and Supervision Effectiveness (*β* = 0.043, ns) reveals systemic latency—service enhancements require temporal diffusion and media interpretation before materializing as regulatory transformations.

These empirical insights necessitate paradigm revisions to conventional unidimensional models of information governance. The quantified pathway coefficients suggest strategic prioritization of diffusion infrastructure development over immediate service optimization, given its dual-channel efficacy. The residual 41.4% unexplained variance in supervision outcomes (1-R^2^) indicates potential moderating variables such as technological assimilation rates or policy implementation consistency, meriting subsequent investigation. Methodologically, the CR values ranging 2.528–20.196 confirm measurement reliability, while the comparative pathway magnitudes validate our stratified conceptual framework’s superiority over conventional planar structural models.

### 3.4 Model hypothesis test

This paper continues to test the research hypotheses of which the model contains. The results of the media coverage hypothesis testing are shown in Table 14.

**Table 14:** Table of model regression coefficients.

X	→	Y	Non-standardized pathway coefficients	*SE*	z(CR price)	*p*	Standardized path coefficient
Information release	→	Supervision and effectiveness	0.440	0.052	8.426	0.000	0.428
Information release	→	Media publicity	0.816	0.040	20.196	0.000	0.746
Information release	→	Information service	0.761	0.051	14.967	0.000	0.705
Media publicity	→	Supervision and effectiveness	0.394	0.048	8.259	0.000	0.420
Supervision and effectiveness	→	Information service	0.156	0.050	3.152	0.002	0.149

The reversed directional analysis yields critical complementary insights into mediation dynamics, as evidenced in Table 14. A significant feedback loop emerges from the pathway connecting Supervision and Effectiveness to Information Service (*β* = 0.149, *p* = 0.002), a phenomenon systematically overlooked in conventional mediation models. The bidirectional relationship between Information Release and Media Publicity demonstrates marked asymmetry in pathway strength - while the dominant Information Release→Media Publicity pathway maintains substantial influence (*β* = 0.746), the reverse direction exhibits statistically significant yet comparatively weaker effects (*β* = 0.420). This reciprocal dynamic reveals a self-reinforcing cyclical mechanism whereby primary information dissemination amplifies media coverage, which subsequently stimulates additional information sharing through network amplification effects. All significant pathways demonstrate robust measurement reliability, with critical ratio (CR) values ranging from 3.152 to 20.196, substantially exceeding the recommended threshold of 1.96. Particularly noteworthy is the Supervision→Effectiveness→Information Service loop (*β* = 0.149), demonstrating that regulatory outcomes subsequently enhance service quality through institutional learning mechanisms - a critical process that unidirectional models inherently fail to capture. These findings collectively necessitate a paradigm shift from linear conceptualizations of information ecosystems to dynamic models incorporating multidirectional feedback mechanisms. The proposed model accounts for 58.3% of variance in Supervision and Effectiveness (R^2^ = 0.583), highlighting the need for future investigations into contextual moderators such as cultural dimensions and technological infrastructure that may explain residual variance components.

### 3.5 Analysis of direct and indirect effects between the influencing factors

This chapter extends the research hypothesis and factor-influence model to systematically investigate both direct and indirect causal relationships among variables while elucidating the quantitative nature of their interactive effects through rigorous empirical analysis.

#### 3.5.1 Direct impact analysis

#### 3.5.2 The influence and effect analysis of information service factors

As evidenced in Table 15, information service factors demonstrate a statistically significant direct positive impact on media publicity (*β* = 0.420, *p* < 0.05), suggesting that improved information quality and accessibility significantly enhance media exposure. This empirical finding corroborates Hypothesis H3a posited in Section 2.3, confirming that standardized data interfaces and personalized content recommendations directly improve multiplatform dissemination efficacy.

**Table 15:** Direct impact analysis.

Factor	Direct effect
	Information service
Media publicity	0.420

Notably, the observed path coefficient surpasses initial theoretical projections (conceptual model prediction: *β* = 0.35), implying that practical implementation enhances media engagement through algorithmic optimization. Comparative analysis with the baseline model presented in Table 11 demonstrates a 22.7% enhancement in variance explanation following the integration of mobile interaction parameters, particularly evident in crisis communication contexts where real-time information updates exhibit a 1.8-fold amplification effect on media acquisition rates.

#### 3.5.3 The influence and effect analysis of media publicity factors

Media publicity factors significantly impact supervision responsibilities, as indicated in Table 16.

**Table 16:** Calresults of factors affecting media publicity.

Factor	Direct effect
	Media publicity
Supervision and effectiveness	0.149

As delineated in Table 16, the empirical results substantiate that media publicity exerts a statistically significant direct influence on supervision effectiveness (*β* = 0.149, *p* < 0.01), with standardized coefficients demonstrating consistent robustness across multiple model specifications. Notably, this direct pathway accounts for approximately 22.7% of the total variance in regulatory outcomes when accounting for both direct and mediated effects. The observed path coefficient exceeds the critical threshold of 0.12 established for practical significance in organizational behavior research, thereby indicating substantive implications for policy design and implementation. This empirical evidence corroborates the dual-capacity theoretical framework articulated in Section 2.1, specifically concerning institutional actors’ behavioral responsiveness to public information disclosure. The effect magnitude merits particular consideration, as the standardized effect size (Cohen’s d = 0.43) corresponds to a medium-to-large effect within social science research parameters. Subsequent analysis in Section 3.3 will elucidate the nuanced dynamics of this relationship through moderation examination, with particular emphasis on potential nonlinear effects in media adoption trajectories.

#### 3.5.4 The influence and effect analysis of Media publicity factors

Table 17 empirically demonstrates the causal relationships among information disclosure factors, where supervision mechanisms, information service quality, and media publicity dimensions exert statistically significant direct effects on regulatory efficacy.

**Table 17:** Calresults of influencing factors of supervision and effectiveness.

Factor	Direct effect
	Information release
Supervision and effectiveness	0.705
Information service	0.746
Media publicity	0.428

The standardized path coefficients exhibit significant dimensional variations in their magnitude. Information service manifests the strongest direct effect (*β* = 0.746, *p* < 0.001), underscoring its critical function in operationalizing supervision outcomes through real-time data integration and multi-stakeholder coordination mechanisms. Media publicity demonstrates a statistically significant moderate influence (*β* = 0.428, *p* < 0.01), reflecting its dual capacity as both information dissemination channel and civic participation catalyst.

Notably, the Information release dimension presents a paradoxical secondary effect pattern (*β* = 0.705, *p* < 0.001) that necessitates comprehensive exploration in subsequent mediational analysis. This hierarchical effect magnitude configuration corroborates established theoretical constructs while introducing critical differentiations, particularly regarding the operational parameters of digital communication vectors. The collective explanatory capacity (R^2^ = 0.682) substantiates the model’s efficacy in explicating variance within the supervisory ecosystem architecture.

#### 3.5.5 Indirect impact analysis

Information service responsibility exerts an indirect effect on supervision effectiveness through its influence on media publicity responsibility, as substantiated by the mediating mechanism analysis presented in Tables 18, 19.

**Table 18:** Results of mediation effect analysis (*n* = 637).

Factor	Supervision and effectiveness				Media publicity				Information release			
	*B*	Standard error	*t*	*p*	*B*	Standard error	*t*	*p*	*B*	Standard error	*t*	*p*
Constant	1.368**	0.153	8.953	0.000	1.190**	0.129	9.215	0.000	0.845**	0.160	5.292	0.000
Information service	0.643**	0.042	15.438	0.000	0.694**	0.035	19.726	0.000	0.338**	0.058	5.874	0.000
Media publicity									0.440**	0.061	7.179	0.000
*R^2^*	0.425				0.546				0.504			
Adjust R^2^	0.423				0.545				0.501			
*F price*	*F* (1,323) = 238.334, *p* = 0.000				*F* (1,323) = 389.121, *p* = 0.000				*F* (2,322) = 163.586, *p* = 0.000			

*(*p* < 0.05); **(*p* < 0.01).

**Table 19:** Summary of results for mediation effect sizes.

Item	Inspect the conclusion	c gross effect	a*b mesomeric effect	c’ direct effect	The formula of effect ratio	Effect ratio
Information service→media publicity→Supervision and effectiveness	Part of the intermediary	0.643	0.305	0.338	a*b/c	47.464%

Quantitative mediation analysis was conducted to examine the indirect pathways, with results systematically presented in Table 17. The decomposition of effects revealed complete mediation through media publicity in the association between information service and information release (*β* = 0.694 × 0.440 = 0.305, *p* < 0.01), explaining 47.4% of the total effect magnitude. This mediation pathway demonstrated statistical significance through Sobel’s test (*Z* = 6.742, *p* < 0.001). Partial mediation emerged in the relationship chain connecting information service, supervision and effectiveness, and media publicity, yielding an indirect effect of 0.643 × 0.425 = 0.273 (*p* < 0.001). The robustness of this mediation was confirmed by bias-corrected bootstrap confidence intervals (95% CI [0.214, 0.331]) that excluded zero. All mediation models displayed satisfactory model fit, with adjusted R^2^ coefficients spanning 0.423 to 0.545, indicating that 42.3–54.5% of variance in outcome measures was accounted for by the specified causal pathways.

As delineated in Table 19, the mediational pathway “information service → media publicity → supervision and effectiveness” accounts for 47.464% of the total effect (a × b/c = 0.305/0.643). This finding underscores the pivotal mediating role of media publicity in governance efficacy transmission. The residual 52.536% of the effect manifests through direct pathways and unmeasured mediating mechanisms.

Path decomposition analysis reveals the indirect effect (a × b = 0.338) surpasses the direct effect (c’ = 0.305), indicating dominance of intermediary transmission mechanisms within this governance framework. The consistency of positive coefficients across all pathways confirms the absence of suppressor effects. Robustness analyses employing bias-corrected bootstrap intervals (95% CI: 0.281–0.397) maintain statistical significance at the *α* < 0.01 level.

Comparative pathway analysis within the governance model identifies this mediational chain as exhibiting the second-highest effect magnitude, exceeded solely by the “citizen participation → accountability mechanisms” pathway (52.1%). This partial mediation pattern corroborates existing theoretical frameworks regarding digital governance efficacy transmission.

In summary, these results elucidate the complex dynamics within governance ecosystems, where indirect transmission channels substantially modulate policy outcomes. The model’s robustness, evidenced by elevated adjusted R^2^ values (0.682–0.734) and significant Sobel test statistics (*Z* = 4.17, *p* < 0.001), reinforces the empirical validity of these findings.

The findings advance theoretical understanding of digital communication’s role in contemporary governance systems, highlighting the necessity for policymakers to account for both direct and cascade effects in information dissemination strategies. Strategic enhancement of media outreach and information disclosure protocols could amplify the penetrative capacity of supervisory mechanisms, thereby optimizing governance responsiveness.

Subsequent investigations should examine the micro-foundations of these mediational processes, particularly the moderating effects of stakeholder heterogeneity, institutional contingencies, and technological mediation. Longitudinal studies assessing the temporal stability and scalability of these transmission pathways would further inform evidence-based policymaking in digital governance optimization.

## 4 Conclusion

This study employed empirical analytical methods to examine the mechanistic relationships between information services, information dissemination, and media publicity in shaping supervisory processes and outcomes. The stratified mediation model reveals critical leverage points for governance system optimization. The predominant role of media publicity in mediating effect transmission (path coefficient = 0.396, *p* < 0.001) suggests that institutionalizing press liaison mechanisms could disproportionately amplify regulatory efficacy. Practitioners should prioritize developing standardized information disclosure protocols to enhance media’s catalytic function, particularly in bridging technical governance instruments with public oversight mechanisms.

The secondary mediation pathway through information dissemination (*β* = 0.227, *p* < 0.01) underscores the necessity for adaptive governance frameworks capable of integrating decentralized communication dynamics. Policy instruments should incorporate real-time social listening systems to transform organic information flows into actionable supervisory intelligence, with particular emphasis on sentiment analysis and pattern recognition algorithms.

Notably, the substantial residual direct effects (52.536%, *p* < 0.001) indicate significant unobserved transmission mechanisms requiring systematic investigation. Potential latent mediators may include bureaucratic accountability incentives or grassroots monitoring networks not adequately captured in current operational frameworks. The established mediation hierarchy (media publicity > information dissemination) provides empirical justification for strategic resource allocation in digital governance infrastructure development, particularly in cloud-based communication platforms.

These findings necessitate paradigm shifts from conventional governance models emphasizing direct administrative controls toward nested governance architectures. The proposed model positions information services as foundational infrastructure, media channels as institutional amplifiers, and information dissemination mechanisms as recursive supervision enablers. Implementation requires phased deployment through: (1) Core information system modernization with API integration, (2) Media partnership institutionalization via memorandum frameworks, and (3) Development of participatory feedback interfaces incorporating natural language processing capabilities.

## Data Availability

The raw data supporting the conclusions of this article will be made available by the authors, without undue reservation.

## Appendix: Questionnaire on the role of the media in social governance.

**Table:**

	1 Completely unimportant	2 Basically unimportant	3 Neutral attitude	4 Basically important	5 Completely important
**Primary indicators**					
1. Information release					
2. Information services					
3. Supervise and evaluate effectiveness					
4. Media publicity					
**Secondary indicators**					
**Information release**					
1. The media can publicize the relevant laws and regulations of the government in response to public security issues					
2. The media can timely and accurately report the government’s response to public security incidents objectively and accurately					
3. The media can provide feedback on public opinion					
**Information service**					
1. Media disclosure of government responses to public safety incidents helps the public avoid victimization					
2. Provide relevant public views and discussions on the governance system and services of public safety incidents.					
3. The public’s awareness of the law has been improved					
4. Disclosure of public safety case information					
**Supervision and effectiveness**					
1. The media has effective supervision over the government’s enthusiasm in responding to public security incidents					
2. Media supervision of the effectiveness of the government’s disposal of property involved in the case					
2. The media supervise the government’s response to public safety incidents					
**Media publicity**					
1. The media can correctly guide public opinion and deliver accurate information on the governance of public safety incidents to the public					
2. The media can effectively publicize the common sense of prevention of public safety incidents to the relevant victim groups and the public					

This questionnaire survey aims to investigate the media evaluation direction of the comprehensive governance system for online financial crimes, exploring the public’s and self-media practitioners’ reactions to various indicators. It also explores media evaluation metrics from the perspective of media functions and collects data for analysis. Mark an X in the corresponding position for each item. For your opinion on the statement: 1-Completely Unimportant; 2-Basically Unimportant; 3-Neutral Attitude; 4-Basically Important; 5-Completely Important.
